# Supplementary material for: Epidemiological characteristics of the B.1.526 SARS-CoV-2 variant
Source: Sci Adv. 2022 Jan 28;8(4):eabm0300. doi: 10.1126/sciadv.abm0300 (PMC8797779; doi:10.1126/sciadv.abm0300)
Supplement: Supplementary file 1 — Tables S1 to S3 Figs. S1 to S3 References [file sciadv.abm0300_sm.pdf]

Science Advances
AAAS

Supplementary Materials for

Epidemiological characteristics of the B.1.526 SARS-CoV-2 variant

Wan Yang*, Sharon K. Greene, Eric R. Peterson, Wenhui Li, Robert Mathes, Laura Graf, Ramona Lall, Scott Hughes, Jade Wang, Anne Fine

*Corresponding author. Email: wy2202@cumc.columbia.edu

Published 28 January 2022, *Sci. Adv.* **8**, eabm0300 (2022)
DOI: 10.1126/sciadv.abm0300

This PDF file includes:

Tables S1 to S3
Figs. S1 to S3
References

Table S1. Prior ranges for the network model-inference system. The prior ranges are similar to Table S1 of Yang et al.(*6*) but include additional parameters in Eqn 1. The spatial, temporal, and age resolution of each parameter or variable, estimated in the model-inference system, is specified in the column "Resolution". Note posterior parameter estimates can extend outside the specified prior ranges.

Parameter/ variable	Symbol	Resolution	Prior range	Source/rationale
Initial exposed	E(t=0)	neighborhood- and age-group specific, estimated for the beginning of the Week of March 1, 2020	300 – 8000 total citywide, scaled by population size for each age group and neighborhood	Large uncertainties, used very wide range
Initial infectious	I(t=0)	neighborhood- and age-group specific, estimated for the beginning of the Week of March 1, 2020	150 – 4000 total citywide, scaled by population size for each age group and neighborhood	Assumed to be half the initial exposed
Initial susceptible	S(t=0)	neighborhood- and age-group specific, estimated for the beginning of the Week of March 1, 2020	$N – E – I$	Assumed all were susceptible except for those initially exposed/infectious
Population size in each age group and neighborhood	N	neighborhood- and age-group specific	N/A	NYC intercensal population estimates for 2018 (*40*)
Citywide transmission rate	β_{city}	Citywide, age-group specific, estimated for each week	[0.5, 1] per day overall; scaled by contact rate for each age group based on contact data from the POLYMOD study(*41*) (averaged across 8 countries)	Based on R_0 estimates of around 1.5-4 for SARS-CoV-2 (*42-44*)
Scaling of neighborhood transmission rate	b_i	neighborhood- and age-group specific, estimated for each week	[0.8, 1.2] for age groups under 65 years; [0.5, 1.5] for age groups 65 or older	Around 1; larger variation for elderly groups based on data
Latency period	Z	Citywide, age-group specific, estimated for each week	[2, 5] days	Incubation period: 5.2 days (95% CI: 4.1, 7) (*42*); latency period is likely shorter than the incubation period

Infectious period	*D*	Citywide, age-group specific, estimated for each week	[2, 5] days	Time from symptom onset to hospitalization: 3.8 days (95% CI: 0, 12.0) in China,(*45*) plus 1-2 days viral shedding before symptom onset. We did not distinguish symptomatic/asymptomatic infections.
Immunity period	*L*	Citywide, age-group specific, estimated for each week	[2.5, 3.5] years	Based on estimated immunity period for endemic human coronaviruses (see Appendix of Yang et al.(*6*))
Multiplicative factor for mobility; see Yang et al.(*6*) for detail	m_1	Citywide, age-group specific, estimated for each week	[1, 2] for <1 year; [0.5, 1.5] for three age groups 1-24 years; [0.1, 1.5] for age group 25-44; [1, 2.5] for age groups 45 or older	Initial model testing showed transmission rates for younger age groups were more sensitive to changes in mobility whereas the two oldest age groups were not sensitive to mobility. For age groups with contact rates lower than the average (based on the POLYMOD study (*41*)), we raised the diagonal elements in the mobility matrix to the power of the relative contact rate (<1) to account for insensitivity of transmission rate in these age groups to mobility.
Multiplicative factor for neighborhood connectivity; see Yang et al.(*6*) for detail	m_2	Citywide, age-group specific, estimated for each week	[0.5, 2]	Likely around 1 but with large uncertainties
Mean of time from viral shedding to diagnosis; see Yang et al.(*6*) for detail	T_m	Citywide, age-group specific, estimated for each week	[3, 8] days	From a few days to a week from symptom onset to diagnosis,(*45*) plus 1-2 days of viral shedding (being infectious) before symptom onset

Standard deviation (SD) of time from viral shedding to diagnosis; see Yang et al.(*6*) for detail	T_{sd}	Citywide, age-group specific, estimated for each week	[1, 3] days	To allow variation in time to diagnosis
Infection-detection rate; see Yang et al.(*6*) for detail	r	Citywide, age-group specific, estimated for each week	Starting from [0.001, 0.05] at time 0 and allowed to increase over time using space re-probing(*46*)	Large uncertainties
Infection fatality risk (IFR); see Yang et al.(*6*) for detail		Citywide, age-group specific, estimated for each week	[5, 15]×10^{-5} for ages under 25; [5, 15]×10^{-4} for ages 25-44; [5, 15]×10^{-3} for ages 45-64; [0.01, 0.1] for ages 65-74; [0.02, 0.2] for ages 75+;	Based on previous estimates(*47*) but extend to have wider ranges
Time from diagnosis to death; see Yang et al.(*6*) for detail		Citywide	Gamma distribution with mean of 9.36 days and SD of 9.76 days	Based on *n*=15,686 COVID-19 confirmed deaths in NYC as of May 17, 2020.
ED consultation rate (EDR)		Citywide, age-group specific, estimated for each week	[0.001, 0.02] for ages under 25; [0.003, 0.03] for ages 25-44; [0.006, 0.06] for ages 45-64; [0.01, 0.15] for ages 65-74; [0.02, 0.25] for ages 75+;	Based on the ratio of total ED visits and estimated infections during March – Dec 2020
Time-from-infectiousness-to-ED or hospitalization		Citywide, for all ages	Gamma distribution with mean of [5, 7] days and SD of [2, 4] days	

Table S2. Initial conditions used to simulate co-circulation of different variants in the multi-variant, age-structured model. To partially account for changing infection-detection rate, ED-consultation rate (EDR) and IFR, for these three parameters, we used the model-inference estimates averaged over the entire simulation period (i.e. Nov 2020 – April 2021). For the initial transmission rate (for the preexisting non-VOC/VOI variants), we used the model-inference estimates averaged over the week of 10/25/2020 – the week of 11/7/2020 (i.e. the 3 weeks around the start of simulation). For the rest of model state variables and parameters, we used model-inference estimates made at the week of 10/25/2020. For B.1.1.7, we used the following ranges based on estimates from Yang and Shaman (*32*): 40.3 – 52.3% higher transmissibility (related to estimates for the preexisting non-VOC/VOI variants listed below) and 0 – 10% immune escape; for comparison, contact tracing data from the UK showed that B.1.1.7 was 30-50% more infectious.(*37*) For B.1.427/ B.1.429, we used the following ranges based on estimates from Deng et al.(*38*): 16 – 24% higher transmissibility and 0-10% immune escape (vs. 21.4 – 27.8% increase in transmission rate in Deng et al.(*38*) without accounting for changes in immunity due to potential immune escape).

variant	parameter	lower bound	upper bound
B.1.526	Low initial prevalence (%)	0.5	2.5
B.1.526	High initial prevalence (%)	1.5	3.5
B.1.526	Wide initial prevalence (%)	0.5	3.5
B.1.1.7	Increase in transmission rate	0.403	0.5227
B.1.1.7	Immune escape	0	0.1
B.1.427/9	Increase in transmission rate	0.16	0.24
B.1.427/9	Immune escape	0	0.1
non-VOC/VOI	Travel-related importation ε_i	Nominally set to 1 per week for the entire city (N = 8.4 million people)	
B.1.526	Travel-related importation ε_i	Set to 0 as it emerged locally	
B.1.1.7	Travel-related importation ε_i	For the entire city (N = 8.4 million), set to 1 per 2 days for 11/1 – 11/15/20 to reflect lower initial seeding, 1.5 per day for 11/16 -12/31/20 to reflect higher seeding during the holidays, and 2 per day for 1/1 – 4/30/21 to reflect higher seeding due to increases in these variants in the US. Same settings were used for B.1.1.7 and B.1.427/9, because once local transmission is established, travel-related importation plays a nominal role.	
B.1.427/9	Travel-related importation ε_i		
non-VOC/VOI	β_{11} (per day, same below)	0.14	0.21
non-VOC/VOI	β_{22}	0.15	0.2
non-VOC/VOI	β_{33}	0.16	0.22

non-VOC/VOI	β_{44}	0.16	0.23
non-VOC/VOI	β_{55}	0.24	0.36
non-VOC/VOI	β_{66}	0.17	0.25
non-VOC/VOI	β_{77}	0.17	0.22
non-VOC/VOI	β_{88}	0.19	0.25
non-VOC/VOI	β_{12}	0.071	0.1
non-VOC/VOI	β_{13}	0.018	0.027
non-VOC/VOI	β_{14}	0.0074	0.011
non-VOC/VOI	β_{15}	0.023	0.034
non-VOC/VOI	β_{16}	0.011	0.015
non-VOC/VOI	β_{17}	0.0075	0.011
non-VOC/VOI	β_{18}	0.0052	0.0075
non-VOC/VOI	β_{21}	0.074	0.1
non-VOC/VOI	β_{23}	0.019	0.026
non-VOC/VOI	β_{24}	0.0078	0.011
non-VOC/VOI	β_{25}	0.024	0.033
non-VOC/VOI	β_{26}	0.011	0.015
non-VOC/VOI	β_{27}	0.0079	0.011
non-VOC/VOI	β_{28}	0.0054	0.0074
non-VOC/VOI	β_{31}	0.02	0.028
non-VOC/VOI	β_{32}	0.02	0.028
non-VOC/VOI	β_{34}	0.0098	0.014
non-VOC/VOI	β_{35}	0.013	0.018
non-VOC/VOI	β_{36}	0.0072	0.01
non-VOC/VOI	β_{37}	0.0051	0.0071
non-VOC/VOI	β_{38}	0.0066	0.0091
non-VOC/VOI	β_{41}	0.0085	0.013
non-VOC/VOI	β_{42}	0.0085	0.013
non-VOC/VOI	β_{43}	0.013	0.02
non-VOC/VOI	β_{45}	0.014	0.021
non-VOC/VOI	β_{46}	0.01	0.015
non-VOC/VOI	β_{47}	0.0037	0.0054
non-VOC/VOI	β_{48}	0.0069	0.01
non-VOC/VOI	β_{51}	0.11	0.17
non-VOC/VOI	β_{52}	0.11	0.17
non-VOC/VOI	β_{53}	0.092	0.14
non-VOC/VOI	β_{54}	0.073	0.11
non-VOC/VOI	β_{56}	0.07	0.11
non-VOC/VOI	β_{57}	0.042	0.064
non-VOC/VOI	β_{58}	0.041	0.062

non-VOC/VOI	β_{61}	0.053	0.076
non-VOC/VOI	β_{62}	0.053	0.076
non-VOC/VOI	β_{63}	0.043	0.063
non-VOC/VOI	β_{64}	0.051	0.074
non-VOC/VOI	β_{65}	0.053	0.076
non-VOC/VOI	β_{67}	0.058	0.084
non-VOC/VOI	β_{68}	0.052	0.076
non-VOC/VOI	β_{71}	0.032	0.041
non-VOC/VOI	β_{72}	0.032	0.041
non-VOC/VOI	β_{73}	0.022	0.028
non-VOC/VOI	β_{74}	0.0096	0.012
non-VOC/VOI	β_{75}	0.023	0.029
non-VOC/VOI	β_{76}	0.032	0.042
non-VOC/VOI	β_{78}	0.066	0.085
non-VOC/VOI	β_{81}	0.028	0.036
non-VOC/VOI	β_{82}	0.028	0.036
non-VOC/VOI	β_{83}	0.03	0.038
non-VOC/VOI	β_{84}	0.022	0.028
non-VOC/VOI	β_{85}	0.027	0.034
non-VOC/VOI	β_{86}	0.047	0.06
non-VOC/VOI	β_{87}	0.073	0.093
all	Z_1 (days, same below)	2.9	4
all	Z_2	3.3	4.3
all	Z_3	3.4	4.4
all	Z_4	3.4	4.4
all	Z_5	3.5	4.4
all	Z_6	3.5	4.5
all	Z_7	3.3	4.2
all	Z_8	3.3	4.2
all	D_1	2.1	2.9
all	D_2	2.7	3.6
all	D_3	3.2	4.1
all	D_4	3.4	4.4
all	D_5	3.1	4.1
all	D_6	3.2	4.2
all	D_7	2.8	3.7
all	D_8	2.5	3.3
all	IFR_1	3.10E-05	5.00E-05
all	IFR_2	3.00E-05	4.90E-05
all	IFR_3	3.10E-05	4.90E-05

all	IFR_4	3.00E-05	4.90E-05
all	IFR_5	3.00E-04	0.00048
all	IFR_6	0.0029	0.004
all	IFR_7	0.013	0.016
all	IFR_8	0.046	0.055
all	EDR_1	0.0087	0.013
all	EDR_2	0.0059	0.0084
all	EDR_3	0.0023	0.0033
all	EDR_4	0.0046	0.006
all	EDR_5	0.01	0.012
all	EDR_6	0.019	0.023
all	EDR_7	0.031	0.039
all	EDR_8	0.057	0.07
all	*Infection detection rate,* r_1	0.14	0.19
all	*Infection detection rate,* r_2	0.21	0.26
all	*Infection detection rate,* r_3	0.23	0.29
all	*Infection detection rate,* r_4	0.28	0.34
all	*Infection detection rate,* r_5	0.39	0.47
all	*Infection detection rate,* r_6	0.36	0.42
all	*Infection detection rate,* r_7	0.33	0.41
all	*Infection detection rate,* r_8	0.34	0.41

Table S3. Estimated IFR for different variants and changes compared to the baseline risk estimated for preexisting variants during Oct – Dec 2020, using Eqn 3.

Age	IFR, baseline (%)	IFR, B.1.526 (%)	Changes, B.1.526 (%)	Model fit, R^2
<25	0.004 (0.0021, 0.0059)	0.004 (0.0038, 0.0041)	-0.03 (-3.8, 3.8)	1
25-44	0.04 (0.021, 0.059)	0.035 (0.031, 0.038)	-12 (-21, -3.1)	0.97
45-64	0.29 (0.15, 0.44)	0.42 (0.24, 0.59)	43 (-17, 100)	0.67
65-74	1 (0.57, 2.5)	1.5 (0.53, 2.5)	46 (-50, 140)	0.46
75+	4.1 (2.2, 6.3)	6.1 (5.1, 7)	47 (25, 69)	0.94
all	0.35 (0.2, 0.58)	0.5 (0.4, 0.61)	43 (13, 73)	0.89

Fig S1. Estimated infection-detection rate by age group. Red lines show the estimated median infection-detection rate with surrounding areas indicating the 50% (darker color) and 95% (lighter color) CrI. For comparison, the grey bars show the number of cases reported for each week from the week of Oct 4, 2020 to Apr 25, 2021. Labels of x-axis show the week starts (mm/dd/yy).

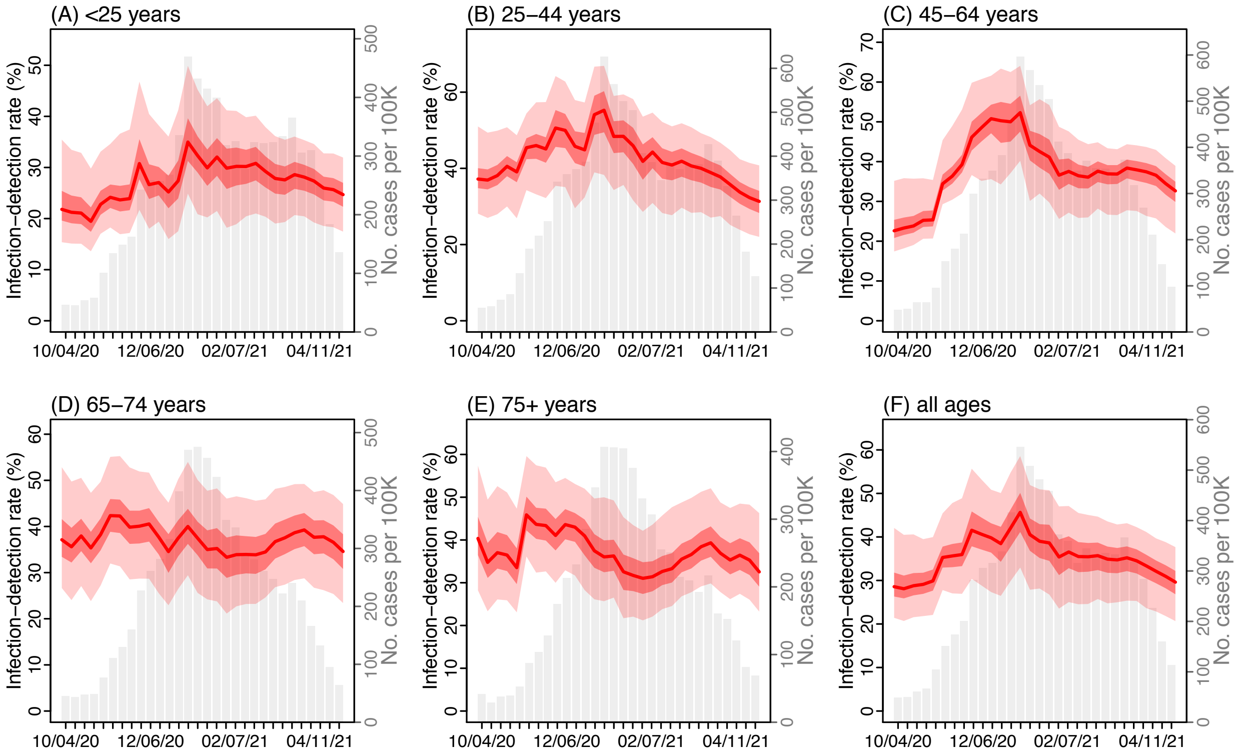

Fig S2. Model-fit by age group. Boxes show model estimates (thick horizontal lines and box edges show the median, 25th, and 75th percentiles; vertical lines extending from each box show 95% Crl) and red dots show corresponding.

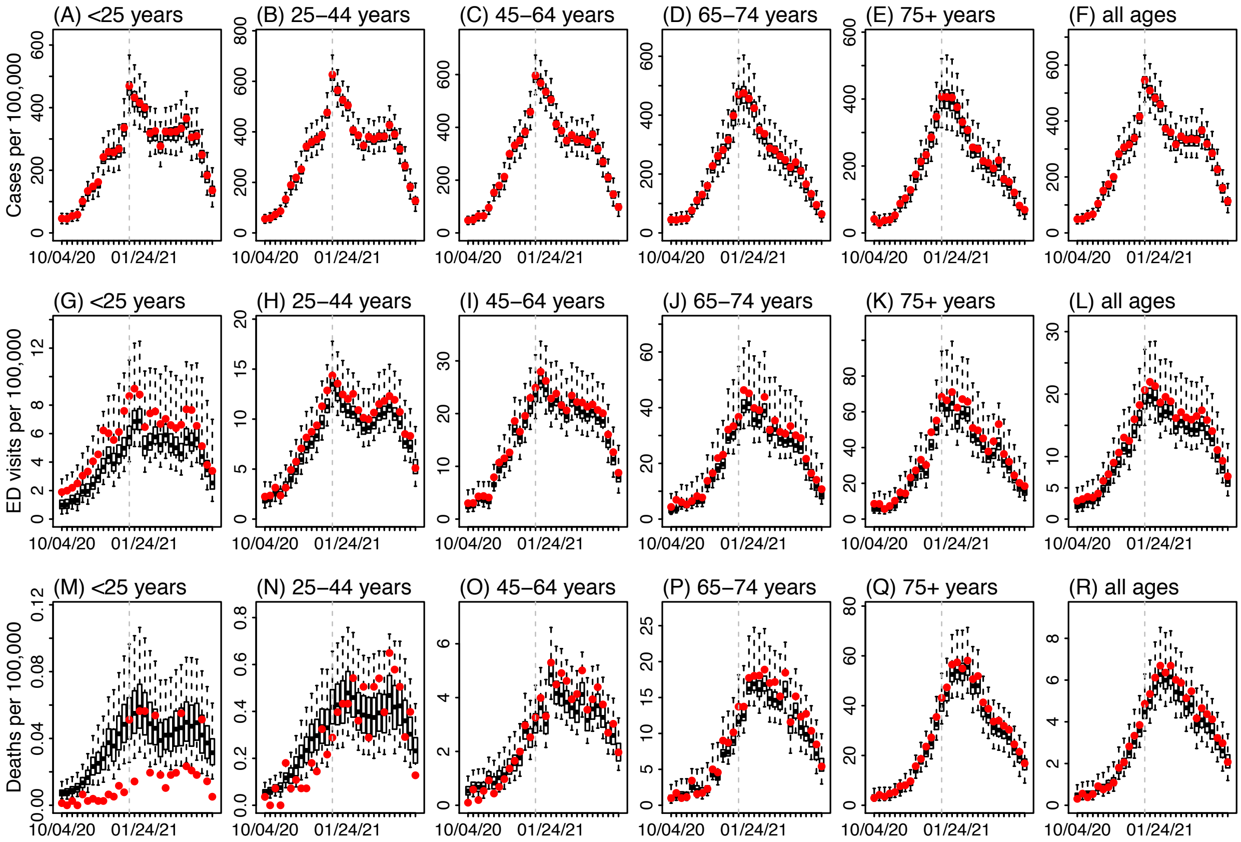

Fig S3. Estimated cumulative infection rates by age group. Thick horizontal lines and box edges show the median, 25th, and 75th percentiles; vertical lines extending from each box show 95% CrI.

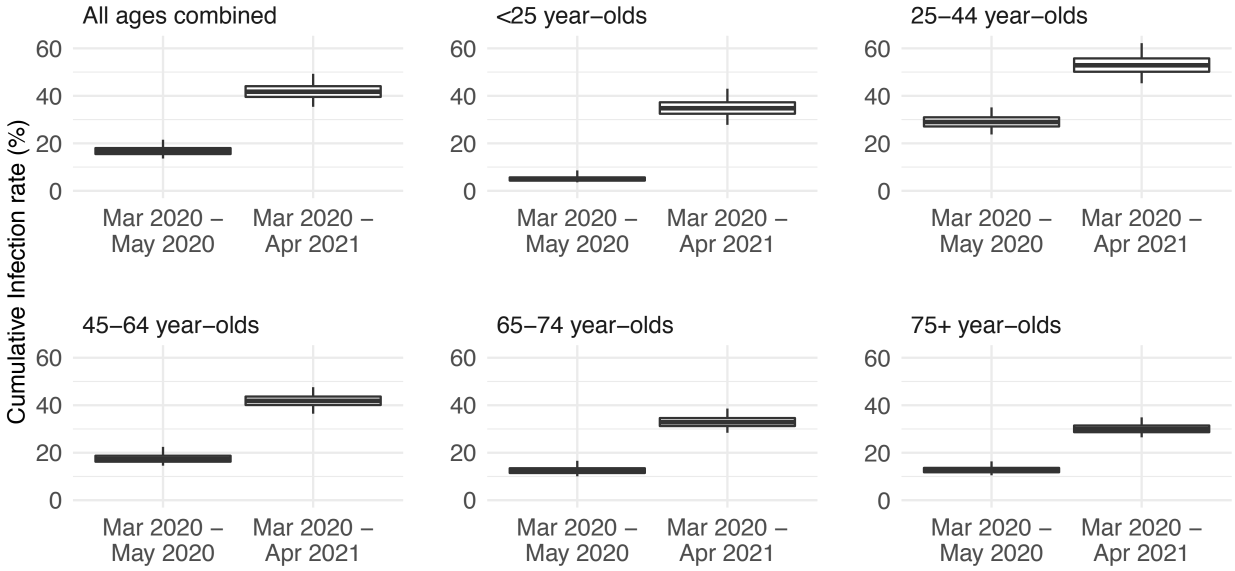

REFERENCES AND NOTES

1. World Health Organization, Tracking SARS-CoV-2 variants (2021); https://www.who.int/en/activities/tracking-SARS-CoV-2-variants/.

2. M. K. Annavajhala, H. Mohri, P. Wang, M. Nair, J. E. Zucker, Z. Sheng, A. Gomez-Simmonds, A. L. Kelley, M. Tagliavia, Y. Huang, T. Bedford, D. D. Ho, A.-C. Uhlemann, A Novel and Expanding SARS-CoV-2 Variant, B.1.526, Identified in New York. *medRxiv*, 2021.2002.2023.21252259 (2021).

3. A. P. West Jr., J. O. Wertheim, J. C. Wang, T. I. Vasylyeva, J. L. Havens, M. A. Chowdhury, E. Gonzalez, C. E. Fang, S. S. Di Lonardo, S. Hughes, J. L. Rakeman, H. H. Lee, C. O. Barnes, P. N. P. Gnanapragasam, Z. Yang, C. Gaebler, M. Caskey, M. C. Nussenzweig, J. R. Keeffe, P. J. Bjorkman, Detection and characterization of the SARS-CoV-2 lineage B.1.526 in New York. *bioRxiv*, 2021.2002.2014.431043 (2021).

4. C. N. Thompson, S. Hughes, S. Ngai, J. Baumgartner, J. C. Wang, E. McGibbon, K. Devinney, E. Luoma, D. Bertolino, C. Hwang, K. Kepler, C. Del Castillo, M. Hopkins, H. Lee, A. K. DeVito, J. L. Rakeman, A. D. Fine, Rapid emergence and epidemiologic characteristics of the SARS-CoV-2 B.1.526 variant—New York City, New York, January 1-April 5, 2021. *MMWR Morb. Mortal. Wkly. Rep.* **70**, 712–716 (2021).

5. Global Initiative on Sharing All Influenza Data (GISAID), GISAID (2021).

6. W. Yang, S. Kandula, M. Huynh, S. K. Greene, G. van Wye, W. Li, H. T. Chan, E. McGibbon, A. Yeung, D. Olson, A. Fine, J. Shaman, Estimating the infection-fatality risk of SARS-CoV-2 in New York City during the spring 2020 pandemic wave: A model-based analysis. *Lancet Infect. Dis.* **21**, 203–212 (2021).

7. N. G. Davies, C. I. Jarvis; CMMID COVID-19 Working Group, W. J. Edmunds, N. P. Jewell, K. Diaz-Ordaz, R. H. Keogh, Increased mortality in community-tested cases of SARS-CoV-2 lineage B.1.1.7. *Nature* **593**, 270–274 (2021).

8. Centers for Disease Control and Prevention, SARS-CoV-2 Variant Classifications and Definitions (2021); https://www.cdc.gov/coronavirus/2019-ncov/variants/variant-info.html.

9. New and Emerging Respiratory Virus Threats Advisory Group, "Minutes of the NERVTAG COVID-19 FortiethMeeting: 11 December 2020" (2020).

10. New and Emerging Respiratory Virus Threats Advisory Group, "Minutes of the extraordinary meeting of NERVTAG COVID-19 and SPI-M on SARS-CoV-2 variants: 21 December 2020" (2020).

11. New and Emerging Respiratory Virus Threats Advisory Group, "NERVTAG/SPI-M Extraordinary meeting on SARS-CoV-2 variant of concern 202012/01 (variant B.1.1.7)" (2020).

12. E. Volz, S. Mishra, M. Chand, J. C. Barrett, R. Johnson, L. Geidelberg, W. R. Hinsley, D. J. Laydon, G. Dabrera, Á. O'Toole, R. Amato, M. Ragonnet-Cronin, I. Harrison, B. Jackson, C. V. Ariani, O. Boyd, N. J. Loman, J. T. McCrone, S. Gonçalves, D. Jorgensen, R. Myers, V. Hill, D. K. Jackson, K. Gaythorpe, N. Groves, J. Sillitoe, D. P. Kwiatkowski; The COVID-19 Genomics UK (COG-UK) consortium, S. Flaxman, O. Ratman, S. Bhatt, S. Hopkins, A. Gandy, A. Rambaut, N. M. Ferguson, Transmission of SARS-CoV-2 Lineage B.1.1.7 in England: Insights from Linking Epidemiological and Genetic Data. *medRxiv*, 2020.2012.2030.20249034 (2021).

13. National Institutes of Health, COVID-19 Treatment Guildlines: Anti-SARS-CoV-2 Monoclonal Antibodies (2021); https://www.covid19treatmentguidelines.nih.gov/therapies/anti-sars-cov-2-antibody-products/anti-sars-cov-2-monoclonal-antibodies/.

14. Centers for Disease Control and Prevention, COVID-19: Treatment Considerations for Healthcare Providers (2021); https://www.cdc.gov/coronavirus/2019-ncov/variants/variant-info.html.

15. T. N. Starr, A. J. Greaney, A. Addetia, W. W. Hannon, M. C. Choudhary, A. S. Dingens, J. Z. Li, J. D. Bloom, Prospective mapping of viral mutations that escape antibodies used to treat COVID-19. *Science* **371**, 850–854 (2021).

16. A. J. Greaney, T. N. Starr, P. Gilchuk, S. J. Zost, E. Binshtein, A. N. Loes, S. K. Hilton, J. Huddleston, R. Eguia, K. H. D. Crawford, A. S. Dingens, R. S. Nargi, R. E. Sutton, N. Suryadevara, P. W. Rothlauf, Z. Liu, S. P. J. Whelan, R. H. Carnahan, J. E. Crowe Jr., J. D. Bloom, Complete mapping of mutations to the SARS-CoV-2 spike receptor-binding domain that escape antibody recognition. *Cell Host Microbe* **29**, 44–57.e49 (2021).

17. N. L. Washington, K. Gangavarapu, M. Zeller, A. Bolze, E. T. Cirulli, K. M. S. Barrett, B. B. Larsen, C. Anderson, S. White, T. Cassens, S. Jacobs, G. Levan, J. Nguyen, J. M. Ramirez III, C. Rivera-Garcia, E. Sandoval, X. Wang, D. Wong, E. Spencer, R. Robles-Sikisaka, E. Kurzban, L. D. Hughes, X. Deng, C. Wang, V. Servellita, H. Valentine, P. De Hoff, P. Seaver, S. Sathe, K. Gietzen, B. Sickler, J. Antico, K. Hoon, J. Liu, A. Harding, O. Bakhtar, T. Basler, B. Austin, D. M. Cannell, M. Isaksson, P. G. Febbo, D. Becker, M. Laurent, E. M. Donald, G. W. Yeo, R. Knight, L. C. Laurent, E. de Feo, M. Worobey, C. Y. Chiu, M. A. Suchard, J. T. Lu, W. Lee, K. G. Andersen, Emergence and rapid transmission of SARS-CoV-2 B.1.1.7 in the United States. *Cell* **184**, 2587–2594.e7 (2021).

18. A. J. Greaney, A. N. Loes, K. H. D. Crawford, T. N. Starr, K. D. Malone, H. Y. Chu, J. D. Bloom, Comprehensive mapping of mutations in the SARS-CoV-2 receptor-binding domain that affect recognition by polyclonal human plasma antibodies. *Cell Host Microbe* **29**, 463–476.e6 (2021).

19. Z. Liu, L. A. VanBlargan, L.-M. Bloyet, P. W. Rothlauf, R. E. Chen, S. Stumpf, H. Zhao, J. M. Errico, E. S. Theel, M. J. Liebeskind, B. Alford, W. J. Buchser, A. H. Ellebedy, D. H. Fremont, M. S. Diamond, S. P. J. Whelan, Identification of SARS-CoV-2 spike mutations that attenuate monoclonal and serum antibody neutralization. *Cell Host Microbe* **29**, 477–488.e4 (2021).

20. W. T. Harvey, A. M. Carabelli, B. Jackson, R. K. Gupta, E. C. Thomson, E. M. Harrison, C. Ludden, R. Reeve, A. Rambaut, COVID-19 Genomics UK (COG-UK) Consortium, S. J.

Peacock, D. L. Robertson, SARS-CoV-2 variants, spike mutations and immune escape. *Nat. Rev. Microbiol.* **19**, 409–424 (2021).

21. Centers for Disease Control and Prevention, National Notifiable Diseases Surveillance System (NNDSS) – Coronavirus Disease 2019 (COVID-19) (2021); https://ndc.services.cdc.gov/conditions/coronavirus-disease-2019-covid-19/.

22. New York City Department of Health and Mental Hygiene, Defining Confirmed and Probable Cases and Deaths (2020); https://www1.nyc.gov/site/doh/covid/covid-19-data.page.

23. NewYork City Department of Health and Mental Hygiene, NYC UHF 42 Neighborhoods, http://a816-dohbesp.nyc.gov/IndicatorPublic/EPHTPDF/uhf42.pdf.

24. R. Lall, J. Abdelnabi, S. Ngai, H. B. Parton, K. Saunders, J. Sell, A. Wahnich, D. Weiss, R. W. Mathes, Advancing the use of emergency department syndromic surveillance data, New York City, 2012-2016. *Public Health Rep.* **132**, 23s–30s (2017).

25. SafeGraph, Weekly Patterns: Foot Traffic Data To Understand The COVID-19 Pandemic (2020); https://www.safegraph.com/weekly-foot-traffic-patterns.

26. New York City Department of Health and Mental Hygiene, NYC Coronavirus Disease 2019 (COVID-19) Data (2020); https://github.com/nychealth/coronavirus-data.

27. New york City Department of Health and Mental Hygiene, Variants (2021); https://github.com/nychealth/coronavirus-data/tree/master/variants.

28. F. P. Polack, S. J. Thomas, N. Kitchin, J. Absalon, A. Gurtman, S. Lockhart, J. L. Perez, G. Pérez Marc, E. D. Moreira, C. Zerbini, R. Bailey, K. A. Swanson, S. Roychoudhury, K. Koury, P. Li, W. V. Kalina, D. Cooper, R. W. Frenck Jr., L. L. Hammitt, Ö. Türeci, H. Nell, A. Schaefer, S. Ünal, D. B. Tresnan, S. Mather, P. R. Dormitzer, U. Şahin, K. U. Jansen, W. C. Gruber; C4591001 Clinical Trial Group, Safety and efficacy of the BNT162b2 mRNA Covid-19 vaccine. *New Engl. J. Med.* **383**, 2603–2615 (2020).

29. L. R. Baden, H. M. el Sahly, B. Essink, K. Kotloff, S. Frey, R. Novak, D. Diemert, S. A. Spector, N. Rouphael, C. B. Creech, J. McGettigan, S. Khetan, N. Segall, J. Solis, A. Brosz, C. Fierro, H. Schwartz, K. Neuzil, L. Corey, P. Gilbert, H. Janes, D. Follmann, M. Marovich, J. Mascola, L. Polakowski, J. Ledgerwood, B. S. Graham, H. Bennett, R. Pajon, C. Knightly, B. Leav, W. Deng, H. Zhou, S. Han, M. Ivarsson, J. Miller, T. Zaks, COVE Study Group, Efficacy and safety of the mRNA-1273 SARS-CoV-2 vaccine. *N. Engl. J. Med.* **384**, 403–416 (2021).

30. E. J. Haas, F. J. Angulo, J. M. McLaughlin, E. Anis, S. R. Singer, F. Khan, N. Brooks, M. Smaja, G. Mircus, K. Pan, J. Southern, D. L. Swerdlow, L. Jodar, Y. Levy, S. Alroy-Preis, Impact and effectiveness of mRNA BNT162b2 vaccine against SARS-CoV-2 infections and COVID-19 cases, hospitalisations, and deaths following a nationwide vaccination campaign in Israel: An observational study using national surveillance data. *Lancet* **397**, 1819–1829 (2021).

31. J. L. Anderson, An ensemble adjustment Kalman filter for data assimilation. *Mon. Weather Rev.* **129**, 2884–2903 (2001).

32. W. Yang, J. Shaman, Development of a model-inference system for estimating epidemiological characteristics of SARS-CoV-2 variants of concern. *Nat. Commun.* **12**, 5573 (2021).

33. W. Yang, E. H. Y. Lau, B. J. Cowling, Dynamic interactions of influenza viruses in Hong Kong during 1998-2018. *PLoS Comput. Biol.* **16**, e1007989 (2020).

34. J. R. Gog, B. T. Grenfell, Dynamics and selection of many-strain pathogens. *Proc. Natl. Acad. Sci. U.S.A.* **99**, 17209–17214 (2002).

35. L. J. Abu-Raddad, H. Chemaitelly, A. A. Butt; National Study Group for COVID-19 Vaccination, Effectiveness of the BNT162b2 COVID-19 vaccine against the B.1.1.7 and B.1.351 variants. *N. Engl. J. Med.* **385**, 187–189 (2021).

36. W. F. Garcia-Beltran, E. C. Lam, K. S. Denis, A. D. Nitido, Z. H. Garcia, B. M. Hauser, J. Feldman, M. N. Pavlovic, D. J. Gregory, M. C. Poznansky, A. Sigal, A. G. Schmidt, A John Iafrate, V. Naranbhai, A. B. Balazs, Multiple SARS-CoV-2 variants escape neutralization by vaccine-induced humoral immunity. *Cell* **184**, 2372–2383.e9 (2021).

37. W. Yang, wan-yang/covid_voc_study: covid_voc_study_yang_shaman_2021_NatComm (v1.0). Zenodo (2021); https://doi.org/10.5281/zenodo.5715611

38. Public Health England, Investigation of novel SARS-CoV-2 variant, *Variant of Concern 202012/01: Technical briefing 3* (Public Health England, 2020).

39. X. Deng, M. A. Garcia-Knight, M. M. Khalid, V. Servellita, C. Wang, M. K. Morris, A. Sotomayor-González, D. R. Glasner, K. R. Reyes, A. S. Gliwa, N. P. Reddy, C. S. San Martin, S. Federman, J. Cheng, J. Balcerek, J. Taylor, J. A. Streithorst, S. Miller, G Renuka Kumar, B. Sreekumar, P.-Y. Chen, U. Schulze-Gahmen, T. Y. Taha, J. Hayashi, C. R. Simoneau, S. M. Mahon, P. V. Lidsky, Y. Xiao, P. Hemarajata, N. M. Green, A. Espinosa, C. Kath, M. Haw, J. Bell, J. K. Hacker, C. Hanson, D. A. Wadford, C. Anaya, D. Ferguson, L. F. Lareau, P. A. Frankino, H. Shivram, S. K. Wyman, M. Ott, R. Andino, C. Y. Chiu, Transmission, infectivity, and antibody neutralization of an emerging SARS-CoV-2 variant in California carrying a L452R spike protein mutation. *medRxiv*, 2021.2003.2007.21252647 (2021).

40. New York City Department of Health and Mental Hygiene (NYC DOHMH); https://www.health.ny.gov/health_care/medicaid/redesign/ehr/registry/nycdohmh.htm.

41. J. Mossong, N. Hens, M. Jit, P. Beutels, K. Auranen, R. Mikolajczyk, M. Massari, S. Salmaso, G. S. Tomba, J. Wallinga, J. Heijne, M. Sadkowska-Todys, M. Rosinska, W. J. Edmunds, Social contacts and mixing patterns relevant to the spread of infectious diseases. *PLoS Med.* **5**, e74 (2008).

42. Q. Li, X. Guan, P. Wu, X. Wang, L. Zhou, Y. Tong, R. Ren, K. S. M. Leung, E. H. Y. Lau, J. Y. Wong, X. Xing, N. Xiang, Y. Wu, C. Li, Q. Chen, D. Li, T. Liu, J. Zhao, M. Liu, W. Tu, C. Chen, L. Jin, R. Yang, Q. Wang, S. Zhou, R. Wang, H. Liu, Y. Luo, Y. Liu, G. Shao, H.

Li, Z. Tao, Y. Yang, Z. Deng, B. Liu, Z. Ma, Y. Zhang, G. Shi, T. T. Y. Lam, J. T. Wu, G. F. Gao, B. J. Cowling, B. Yang, G. M. Leung, Z. Feng, Early transmission dynamics in Wuhan, China, of novel coronavirus–infected pneumonia. *New Engl. J. Med.* **382**, 1199–1207 (2020).

43. J. T. Wu, K. Leung, G. M. Leung, Nowcasting and forecasting the potential domestic and international spread of the 2019-nCoV outbreak originating in Wuhan, China: A modelling study, *Lancet* **395**, 689–697 (2020).

44. R. Li, S. Pei, B. Chen, Y. Song, T. Zhang, W. Yang, J. Shaman, Substantial undocumented infection facilitates the rapid dissemination of novel coronavirus (SARS-CoV-2). *Science* **368**, 489–493 (2020).

45. J. Zhang, M. Litvinova, W. Wang, Y. Wang, X. Deng, X. Chen, M. Li, W. Zheng, L. Yi, X. Chen, Q. Wu, Y. Liang, X. Wang, J. Yang, K. Sun, I. M. Longini Jr., M. E. Halloran, P. Wu, B. J. Cowling, S. Merler, C. Viboud, A. Vespignani, M. Ajelli, H. Yu, Evolving epidemiology and transmission dynamics of coronavirus disease 2019 outside Hubei province, China: A descriptive and modelling study. *Lancet Infect. Dis.* **20**, 793–802 (2020).

46. W. Yang, J. Shaman, A simple modification for improving inference of non-linear dynamical systems. *arXiv*, 1403.6804 (2014).

47. R. Verity, L. C. Okell, I. Dorigatti, P. Winskill, C. Whittaker, N. Imai, G. Cuomo-Dannenburg, H. Thompson, P. G. T. Walker, H. Fu, A. Dighe, J. T. Griffin, M. Baguelin, S. Bhatia, A. Boonyasiri, A. Cori, Z. Cucunubá, R. FitzJohn, K. Gaythorpe, W. Green, A. Hamlet, W. Hinsley, D. Laydon, G. Nedjati-Gilani, S. Riley, S. van Elsland, E. Volz, H. Wang, Y. Wang, X. Xi, C. A. Donnelly, A. C. Ghani, N. M. Ferguson, Estimates of the severity of coronavirus disease 2019: A model-based analysis. *Lancet Infect. Dis.* **20**, 669–677 (2020).
